# Polydatin Induces Differentiation and Radiation Sensitivity in Human Osteosarcoma Cells and Parallel Secretion through Lipid Metabolite Secretion

**DOI:** 10.1155/2021/3337013

**Published:** 2021-07-20

**Authors:** Amalia Luce, Stefania Lama, Pilar Chacon Millan, Annalisa Itro, Angelo Sangiovanni, Carlo Caputo, Pasquale Ferranti, Salvatore Cappabianca, Michele Caraglia, Paola Stiuso

**Affiliations:** ^1^Department of Precision Medicine, University of Campania “Luigi Vanvitelli”, 80138 Naples, Italy; ^2^Department of Advanced Medical and Surgical Sciences, University of Campania “Luigi Vanvitelli”, 80138 Naples, Italy; ^3^Institute of Food Sciences-National Research Council, 83100 Avellino, Italy; ^4^Department of Agriculture-University of Naples-Federico II, 80055 Portici, Napoli, Italy; ^5^Biogem Scarl, Institute of Genetic Research, Laboratory of Molecular and Precision Oncology, 83031 Ariano Irpino, Avellino, Italy

## Abstract

Osteosarcoma is a bone cancer characterized by the production of osteoid tissue and immature bone from mesenchymal cells. Osteosarcoma mainly affects long bones (femur is most frequently site) and occur in children and young adults with greater incidence. Here, we investigated the role accomplished by polydatin, a natural antioxidative compound, in promoting osteogenic differentiation alone or after radiation therapy on osteosarcoma cells. In vitro, polydatin significantly induced cell cycle arrest in S-phase and enhanced bone alkaline phosphatase activity. Moreover, the differentiation process was paralleled by the activation of Wnt-*β*-catenin pathway. In combination with radiotherapy, the pretreatment with polydatin promoted a radiosensitizing effect on osteosarcoma cancer cells as demonstrated by the upregulation of osteogenic markers and reduced clonogenic survival of tumor cells. Additionally, we analyzed, by mass spectrometry, the secretion of sphingolipid, ceramides, and their metabolites in osteosarcoma cells treated with polydatin. Overall, our results demonstrate that polydatin, through the secretion of sphingolipids and ceramide, induced osteogenic differentiation, alone and in the presence of ionizing therapy. Future investigations are needed to validate the use of polydatin in clinical practice as a potentiating agent of radiotherapy-induced anticancer effects.

## 1. Introduction

Osteosarcoma (OS) is the most common primary tumor of the bone in children [1]. The incidence rate is about 3.4 cases per million people per year with a higher percentage in males than in females (5.4 vs. 4.0 cases) [2, 3]. Osteosarcoma can affect all bone districts, but in 90% of cases, it concerns long bones and in 50% of cases the knee. Some studies define osteosarcoma as a pathology associated to differentiation, in which genetic and epigenetic changes interrupt the normal differentiation process starting from mesenchymal stem cells. Tumorigenesis may involve a deregulation of proliferative capacity of stem cancer cells that give rise to an aberrant differentiated osteoblastic progeny [4]. Although most osteosarcomas are sporadic, the main risk factors associated with their incidence include the exposure to radiation or chemotherapy, especially alkylating or anthracycline agents, used to treat other cancers. When OS is diagnosed, approximately 15-20% of patients already have macroscopic evidence of metastases, most commonly in the lungs (85-90%), less frequently in the bones (8-10%), and occasionally in the lymph nodes [5]. OS treatment consists of surgical resection, chemotherapy, and radiotherapy [6]. Osteosarcoma is generally considered a radiation resistant tumor; however, radiotherapy is indicated as primary treatment in unresectable or surgically inaccessible lesions, with 60-70 (Gray) Gy total dose [7]. Radiation therapy can also be used as adjuvant treatment in R1 or R2 margins lesions, alone or with chemotherapy, with total doses of 64-68 Gy at high-risk sites, or as palliative treatment on primary site or on metastases in case of pain or high risk of pathological fracture (8-45 Gy total dose) [8]. Polydatin (PD), (trans-piceid 3,5,4′-trihydroxystilbene-3-O-*β*-D-glucopyranoside) is a natural precursor of resveratrol extracted from *Polygonum cuspidatum* plant. PD has a glycosidic ring bound in position C-3 that replaces the hydroxyl group of resveratrol. This modification leads to changes in biological properties of PD that shows better oral absorption and metabolic stability than resveratrol [9]. Moreover, it is absorbed into the cell, using a secondary active transport mechanism, through sodium-dependent glucose transporters (SGLT1) mainly expressed in the stomach and intestines. Previous studies demonstrated a variety of biological functions of PD, including antioxidant properties and protection against ischemia/reperfusion injury, congestive heart failure, endometriosis, and hemorrhagic shock [10–15]. Recent studies have shown that PD is able to improve the survival of mesenchymal stem cells derived from the bone marrow, protecting them from oxidative damage [16]. In addition, PD inhibits the growth of lung cancer and osteosarcoma cells and promotes differentiation of the human colorectal Caco-2 cells [17–19]. Recent studies by Mele et al. demonstrated that PD exerts a significant cytotoxic effect on cancer cells by G6PD inhibition, a rate-limiting enzyme in the pentose phosphate pathway altered in different malignant tumors [20, 21]. The present study is aimed at investigating the role played by PD on the osteosarcoma cells and its effects on osteogenic differentiation process alone or together with radiation therapy.

## 2. Materials and Methods

### 2.1. Drugs and Reagents

Cell culture plastics were purchased from Becton Dickinson (Lincoln Park, NJ). Fetal bovine serum (FBS), phosphate-buffered saline (PBS), L-glutamine, trypsin, and antibiotics were purchased from Gibco (Life Technologies, Carlsbad, CA). Transpolydatin with a purity grade higher than 99% has been provided by Glures S.r.l., Academic Spin-Off of Ca' Foscari University of Venice (Venice, Italy).

### 2.2. Cell Culture

Human osteosarcoma cell lines MG-63 and Saos-2 were available within our research network. MG-63 cells were cultured in RPMI-1640 (Sigma-Aldrich; Merck KGaA, Darmstadt, Germany). Saos-2 cells were grown in Iscove's Modified Dulbecco's Medium (IMDM) (Sigma-Aldrich; Merck KGaA, Darmstadt, Germany). Both media were enriched with 10% heat-inactivated fetal bovine serum (Sigma-Aldrich; Merck KGaA, Darmstadt, Germany), 1% di L-glutamine, and antibiotics (1.0 × 10^4^ *μ*g/mL penicillin and 1.0 × 10^4^ *μ*g/mL streptomycin). All cell lines were grown at 37°C in a 5% CO_2_-humidified atmosphere.

### 2.3. Cell Viability Assay

Saos-2 and MG-63 were seeded in a 96-well plate at appropriate density (3.0 × 10^3^ and 1.6 × 10^3^ cells/well, respectively). After 24 h, cells were exposed to polydatin at increasing concentrations ranging from 0 to 150 *μ*M. Cell viability was evaluated after 24, 48, and 72 hours by MTT assay as previously described [22]. Each experiment was performed at least three times, and the data were expressed as the mean ± SD.

### 2.4. Alkaline Phosphatase (ALP) Activity

ALP activity was used as a marker of osteogenic differentiation in human Saos-2 cells. To discriminate different ALP isoenzymes, heat fraction was performed warming the samples for 15 minutes at 56°C; bone ALP (bALP) is heat-labile, and liver ALP is stable. ALP activity was determined using colorimetric alkaline phosphatase assay kit colorimetric (Abcam ab83369). Each experiment was conducted three times, and the data were expressed as the mean ± SD.

### 2.5. Cell Cycle Analysis

The percentage of cells in G0/G1, S, and G2/M phases was determined by flow cytometry. Briefly, Saos-2 cells were seeded in a 6-well plate at density of 2.0 × 10^5^ cells/plate and after 24 h exposed to 48 *μ*M of polydatin. Then, after 72, 96 h of treatment the cells was detached and washed in PBS 1x, centrifuged, and directly stained in a propidium iodide (PI) solution (50 *μ*g/mL PI in 0.1% sodium citrate, 0.1% NP40, pH 7.4) for 60 min at 4°C in the dark. Flow cytometric analysis was performed using a BD Accuri™ C6 instrument (Becton Dickinson, San Jose, CA, USA). To evaluate cell cycle, PI fluorescence was collected as FL2. Analysis was carried out in triplicate in three separate experiments, and the data were expressed as the mean ± SD.

### 2.6. Immunostaining and Laser Scanning Confocal Microscopy

Saos-2 cells were fixed for 20 min with a 3% (*w*/*v*) paraformaldehyde solution and permeabilized with 0.1% (*w*/*v*) Triton X-100 in PBS at room temperature. To prevent nonspecific interactions of antibodies, cells were treated for 2 h in 5% (*w*/*v*) BSA in phosphate-buffered saline and then were incubated with a specific rabbit Ab raised against anti-*β*-catenin (Alexa Fluor®, BD Pharmingen™) for 2 h at 37°C. After several washes, the slides were mounted using ProLong™ Diamond Antifade Mountant with DAPI. The analyses were performed with a Zeiss LSM 510 microscope equipped with a plan-apochromat objective X 63 (NA 1.4) in oil immersion.

### 2.7. Wound Healing Assay

Saos-2 cells were seeded in 6-well plates under normal culture conditions at the density of 5.0 × 10^5^ cells/well. Once it reached ~90% confluence, the medium was removed and the cell monolayer was scratched across the center using a sterile pipet tip (200 *μ*L) to produce a uniform wound area. Through gently washing with sterile PBS1X, cellular debris was removed from the well and the cells incubated with 48 *μ*M of polydatin. Cell migration was monitored by microscopy using an inverted light microscope (Eclipse TE 300 Nikon, New York, USA), and images were captured (10x magnification) at different time points after the scratch (0–48 h). The images acquired were analyzed quantitatively, using a specific wound healing tool of ImageJ software (Maryland, USA). The percentage of relative wound closure was assessed according to the following equation [23]:

(1) $$\begin{array}{c} Wound\;closure\%=\frac{W_{0}-W_{n}}{W_{0}}\times 100, \end{array}$$

in which *W*_*n*_ is the width of the cut after the treatment time and *W*_0_ is the width of the scratch before treatment.

### 2.8. Colony Formation Assay

Clonogenic assay was used to evaluate the effect of polydatin on the radiation-resistant osteosarcoma cells. Saos-2 cells were seeded in a 6-well plate at density of 1.0 × 10^4^ cells/well and exposed to polydatin (48 *μ*M) for 96 h. Then, cells were irradiated with 0, 2, 4, and 6 Gy doses of X-rays (Siemens Mevatron LINAC). After 10 days, colonies were fixed with 70% ethanol and stained with 0.25% crystal violet (Sigma-Aldrich, St. Louis, MO, USA). The area of colonies was estimated using ImageJ software. Each sample was processed in triplicate, and the data were expressed as the mean ± SD.

### 2.9. Real-Time PCR

Total RNA was extracted using mirVana PARIS kit (Ambion, Life Technologies, Carlsbad, CA, USA) according to the manufacturer's protocol. RNA quality and quantity were measured by NanoDrop ND-1000 microspectrophotometry (Thermo Fisher Scientific, Wilmington, NC, USA). Reverse transcription was performed according to High Capacity cDNA Reverse Transcription Kit (Applied Biosystems, CA, USA) instructions. qPCR results were obtained from the mean of triplicate measurements for osteopontin, Notch1, and Notch2 using ViiA 7 real-time PCR system (Applied Biosystems, CA, USA), normalized for the mRNA levels of the housekeeping reference gene (HPRT1, also amplified in triplicate).

### 2.10. LC-MS/MS Lipid Analysis

Lipid were extracted from samples in chloroform : methanol (2 : 1) [24]. MS analysis was performed by direct injection of lipid extracts using an automatic infusion syringe coupled with a nano-ESI-linear ion trap (LIT) Thermo XL mass spectrometer (Thermo Fisher Scientific, Waltham, MA, USA). Samples were injected (Thomas Scientific, NJ, USA) at a flow rate of 5 *μ*L/min. MS data were scanned in both positive and negative ion modes from 200 to 2000 *m*/*z* mass range. Data-dependent MS/MS spectra were collected from the upon fragmentation using CID activation with 35.0% normalized collision energy, activation Q of 0.25, and activation time of 30 ms. Spectra were processed using the Xcalibur Software 3.1 version (Thermo Fisher Scientific, Waltham, MA, USA).

### 2.11. Statistical Analysis

Statistical analysis was performed using GraphPad Prism (version 6.01, GraphPad Software Inc., San Diego, CA), and values of *p* ≤ 0.05 were considered significant. Differences between the treatment and control groups were calculated using Student's *t*-test.

## 3. Results

### 3.1. Antiviability Effects of Polydatin on Osteosarcoma Cell Lines

**Figure 1:**
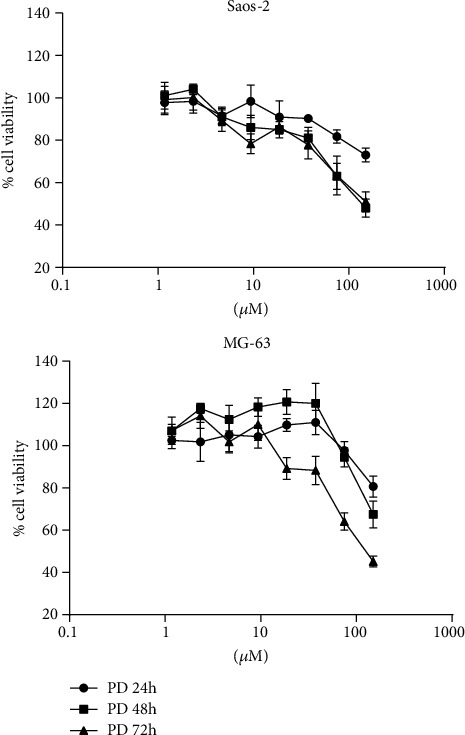
PD effects on osteosarcoma cell line growth. Cell viability was evaluated by MTT assay, after 24, 48, and 72 h of treatment with polydatin in MG-63 and Saos-2 osteosarcoma cell lines.

We evaluated the effect of polydatin on cell viability, *in vitro*, of both MG-63 and Saos-2 human osteosarcoma cell lines. Osteosarcoma cells were treated for 24, 48, and 72 hours with increasing concentrations of polydatin (0-150 *μ*M) and cell viability assessed with MTT assay as reported in Materials and Methods (Figure 1). Polydatin (PD), after 48 hours of treatment, induced an about 25% cell growth inhibition (IC25), in MG-63 and Saos-2 cells at 125 and 48 *μ*M, respectively (Table 1).

Between these two cell lines, Saos-2 cells resemble human osteoblasts because of low proliferation rate, high matrix mineralization ability, and elevated levels of alkaline phosphatase (ALP) activity [25]. In our previous paper, we have demonstrated that polydatin induced a transition from a proliferative morphology to cell-specific differentiated structures in human colon cancer Caco-2 cells [19]. Additionally, ALP in Saos-2 cells can be additionally stimulated by molecules such as dexamethasone that induces an osteoblast-like differentiated phenotype [26]. Therefore, the cellular model chosen appeared to be the most suitable in demonstrating the ability of PD to induce differentiation and sensitize osteosarcoma cells to radiotherapy. All subsequent experiments were performed using Saos-2 cells exposed to polydatin at 48 *μ*M in order to evaluate molecular mechanisms induced by PD alone or in combination with radiation exposure.

### 3.2. Polydatin Induces Cell Cycle Arrest and Enhances Bone Alkaline Phosphatase Activity

**Figure 2:**
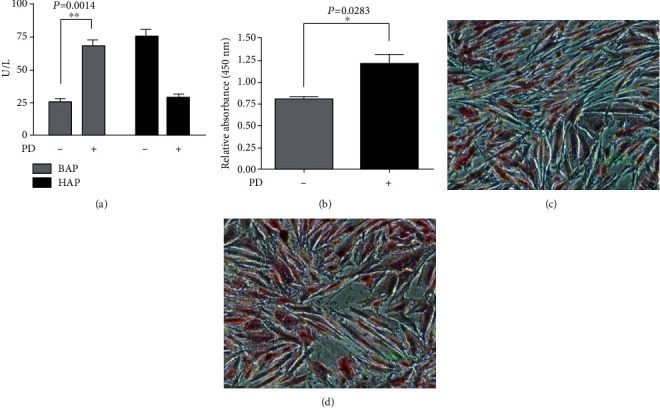
PD increases BAP activity and mineralization: (a) effect of PD on both bone (BAP) and hepatic alkaline phosphatase (HAP) activities in Saos-2 cells. Cells were treated with 48 *μ*M of polydatin and BAP and HAP activities measured using a colorimetric assay. The values are expressed as the means ± SD of three independent experiments; (b) quantitative measurement of the mineralization process after Alizarin Red staining at 405 nm absorbance; images of Alizarin Red staining of (c) untreated Saos-2 cells and (d) cells treated with polydatin.

In order to elucidate whether PD was involved in cell cycle regulation, we analyzed its effects on cell cycle distribution by flow cytometry. Saos-2 cells were treated for 72 and 96 hours with PD and subsequently labeled with propidium iodide for 1 h, and the percentage of cells in each phase of the cell cycle (G0/G1-S-G2/M) was determined. The treatment with PD induced a significant increase in S phase with a concomitant reduction in G2/M phase if compared to the control cells (Table 2). In PD-treated cells, the percentage in S phase increased from 8.3% to 22.5% and in G2/M phase decreased from 59.8% to 47.6%, while the G0/G1 phase remained unaltered if compared to control cells. The cell cycle arrest in S phase is one of the prerequisites for cell differentiation [27]. Therefore, we determined the effect of PD on osteogenic differentiation assessing the bone alkaline phosphatase (BAP) activity. As shown in Figure 2, a significant increase of BAP activity was detected after 96 hours in PD-treated Saos-2 cells compared to untreated cells. On the contrary, the level of hepatic alkaline phosphatase activity (HAP) decreased in PD-treated cells if compared to control cells (Figure 2(a)). In addition, we examined the effects of PD on osteoblast-mediated mineralization by ARed Assay (Figures 2(b)–2(d)).

We found that PD promoted osteoblast-mediated mineralization over the control cells. These findings indicate that PD promoted osteoblast differentiation and mineralization in Saos-2 cells. In addition, after 96 hours, the PD-treatment of Saos-2 cells induced an increase of approximately 20% of extracellular nitric oxide production, compared to the untreated cells (data not shown). Our findings suggest that the reduction in cell viability in Saos-2 cells elicited by PD was associated to the increased level of free NO that might be responsible of cellular differentiation and structural changes. Polydatin redistributes *β*-catenin protein and attenuates migration of osteosarcoma cancer cells.

**Figure 3:**
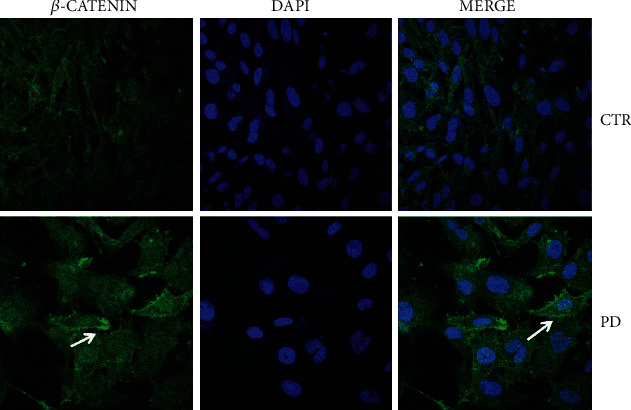
Confocal microscopy images of untreated Saos-2 cells (CTR) and treated with polydatin (PD) at 48 *μ*M for 96 h. The merged images are on the right side: *β*-catenin (green) and DAPI (blue).

Cancer-cell migration is a critical process to create distant metastases. The development and progression of cell motility depend on cytoskeletal modifications, changes in cell-substrate adhesive properties, and alterations in the extracellular matrix [28]. In order to study the impact of PD on the metastatic potential of osteosarcoma cells, we assessed the morphological changes triggered by a sublethal concentrations of PD. We have observed, through confocal microscopy, in PD-treated Saos-2 cells a higher cytoplasm/nucleus ratio and an irregular shape if compared to untreated cells (CTR). Moreover, PD induced, after 96 h, a more compact layer of cells that were adherent to neighboring cells together with plasmatic membrane localization of *β*-catenin (Figure 3), suggesting a less-invasive phenotype.

**Figure 4:**
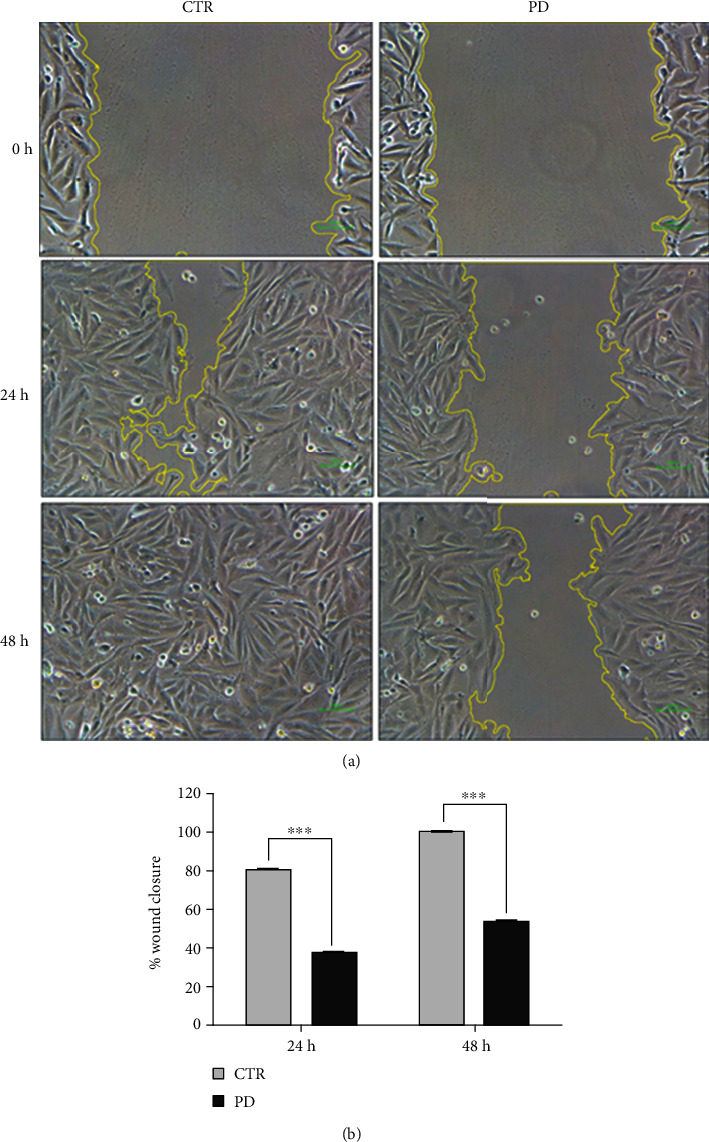
Wound healing assay to determine cell migration. The wound-healing assays were performed at 0, 24, and 48 h in Saos-2 cells treated with polydatin (PD) and untreated cells (CTR). (a) Representative phase contrast microscope images showing the area covered by the cells at 0, 24, and 48 h after wounding (10x magnification). (b) The percentage of relative wound closure was assessed using a specific wound healing tool of ImageJ software (^∗∗∗^*p* < 0.001).

To analyze the capacity of polydatin in inducing the impairment of cancer cell migration, a wound healing assay experiment was performed. A cell-free area was created in a confluent monolayer by removing Saos-2 cells from the area through mechanical damage, and cells were exposed to the polydatin for 24 and 48 hours. As shown in Figures 4(a) and 4(b) at 48 h, control cells had substantially closed the wound, whereas the migration of PD-treated cells was impaired, and the percentage of open wound area was equal to 53.6. Our data clearly show that treatment with PD caused a significant inhibition of cell migration at sublethal concentration in OS cells.

### 3.3. Polydatin Sensitizes Saos-2 Cells to Ionizing Radiation

**Figure 5:**
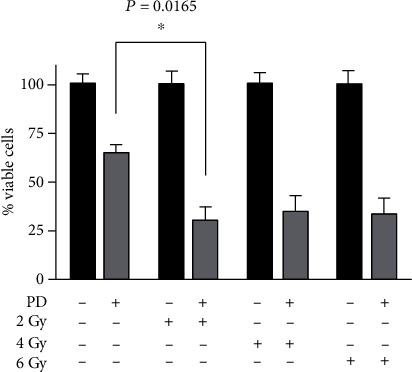
PD effect on cell viability of irradiated Saos-2 cells. Saos-2 cells were pretreated for 96 h with 48 *μ*M polydatin and then exposed to 0, 2, 4, and 6 Gy. The effect of irradiation on cell growth was assessed by Trypan Blue assay after 24 h. The percentage of cell viability of the PD-pretreated cells exposed to 2 Gy was significantly lower than those of the PD-pretreated cells (0 Gy) (^∗^*p* < 0.05).

**Figure 6:**
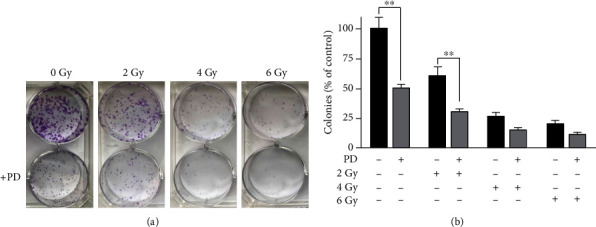
PD effects on colony formation of irradiated Saos-2 cells. PD-pretreated Saos-2 cells were exposed to 0, 2, 4, and 6 Gy of X-irradiation, and crystal violet staining was performed on day 10 after irradiation. (a) Images of clonogenic assay performed in six-well plates, with clones produced by Saos-2 osteosarcoma cells. (b) The area of colonies was estimated using ImageJ software. Data were expressed as the mean ± SD of 3 wells (^∗∗^*p* < 0.01).

It was previously reported that osteosarcoma is associated with poor prognosis due to its high incidence of metastases and chemo and radioresistance. Generally, combination treatment strategies have the aim to potentiate the anticancer activity and/or reduce the therapeutic doses of the anticancer drugs in order to decrease detrimental side effects. To investigate if PD pretreatment could increase the effectiveness of radiotherapy, both cell viability (Figure 5) and colony formation assays (Figure 6) were performed. Saos-2 cells were pretreated with 48 *μ*M of PD for 96 hours and then irradiated with 2, 4, and 6 Gy. The results showed that Saos-2 cells were not sensitive to radiation. In fact, the percentage of viable cells was unchanged compared to the control cell, while PD pretreatment for 96 hours significantly increased the sensitivity of the cells to low radiation dosages (2 Gy) inducing a decrease in viability of about 4- and 2-fold if compared to either untreated or PD-pretreated cells, respectively (Figure 5).

Thereafter, we performed a clonogenic survival assay in order to understand whether the effects of PD on Saos-2 cells may be associated to increased cell radiosensitivity. Saos-2 cells were pretreated for 96 h with 48 *μ*M of PD and then exposed to 0, 2, 4, and 6 Gy radiation, respectively. The cells were cultured for 10 days and colonies stained with crystal violet (Figure 6).

**Figure 7:**
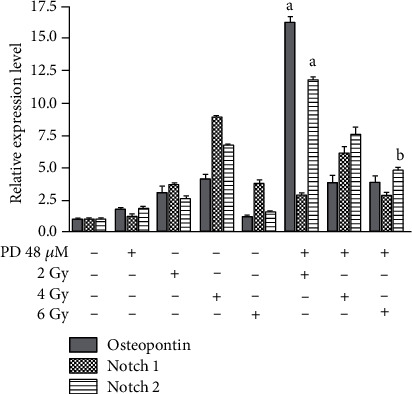
PD effect on relative expression of osteopontin (OPN), Notch1, and Notch2 of irradiated Saos-2 cells. The expression profiles of OPN, Notch1, and Notch2 were determined in the Saos-2 cells without and with PD-pretreatment and subjected to 0, 2, 4, and 6 Gy using a real-time qPCR method ^a^*p* < 0.0001 and ^b^*p* < 0.0003, vs. control represented from the marker values evaluated in cells treated only with 2,4, and 6 Gy.

Saos-2 cells treated with PD showed a significant decrease of cell survival when compared to the control cells (*p* = 0.0018). After exposure to radiation, the PD-pretreated Saos-2 cells showed a decreased (*p* = 0.0059) cell survival when compared to Saos-2 cells exposed to radiation alone. Zhang et al. reported that differentiated cancer cells, after radiotherapy, may reacquire stem cell traits, increased proliferation, and cellular migration [29]. Moreover, in literature, the involvement of Notch pathway during osteogenic differentiation in human osteosarcoma cell line was reported. Activation of Notch2, inducing osteoblast differentiation, not the depression of Notch1, maintained an undifferentiated status [24]. Considering that OS could be induced by disruptions of differentiation of osteoblasts, we have analyzed the mRNA expression level of osteopontin (OPN), a late marker of mesenchymal stem cell differentiation, and Notch1 and Notch2 after the PD and radiation treatment of Saos-2 cells. The results showed a significant increase of OPN and Notch2 mRNA expression level in Saos-2 cells treated with PD in combination with 2 Gy radiation (Figure 7), when compared to the untreated PD cells. Interestingly, in PD-pretreated cells exposed to 2, 4, and 6 Gy radiation, we found a Notch1 decreased expression level compared to the Saos-2 cells submitted at the same radiation doses without PD-pretreatment. The transformation of poorly differentiated Saos-2 cells into highly differentiated cells induced by the PD in combination with radiation could be helpful to induce terminal differentiation of OS cells [30].

### 3.4. Polydatin Modulates the Synthesis and Secretion of Sphingolipids, Ceramides, and Their Metabolites

**Figure 8:**
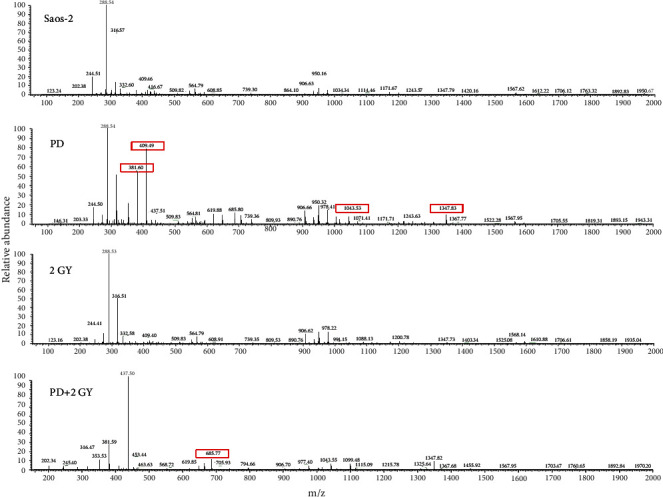
Effect of PD on lipid extracted from media of irradiated Saos-2 cells. Positive ion electrospray mass spectra of lipid molecular species in lipid extracts from the medium of the Saos-2, treated with PD before and after 2 Gy irradiation. Aliquots of chloroform extracts were analyzed directly by electrospray as described in Materials and Methods. Selected peaks are indicated by their *m*/*z* values.

The different lipid species released in the medium of the Saos-2 cells exposed or not to PD, 2 Gy, and PD+2Gy were detected in a positive ionization mode in the range 200–2000 of *m*/*z* (Figure 8). In all mass spectra, even if with different intensities, we detected the molecular peak [M+H]^+^ at 288 *m*/*z*, 316 *m*/*z*, 409 *m*/*z* 906 *m*/*z*, 950 *m*/*z*, and 978 *m*/*z* identified as C17-Sphinganine, dehydrophytosphingosine, LPA (16 : 1), PI (18 : 1 (9Z)/21 : 0), PI-Cer (d20 : 0/26 : 0), and PI (22 : 0/22 : 1), respectively, while the molecular peak [M+H]^+^ at 381 *m*/*z*, 437 *m*/*z*, 619 *m*/*z*, 685 *m*/*z*, 1043 *m*/*z*, and 1347 *m*/*z* identified as Sphinganine-1-P, PE (P-16 : 0), CerP (d18 : 1/16 : 0), Cer (18 : 0/24 : 0 (2OH)), PIM1 (18 : 1/18 : 1), and PIM3 (18 : 2/18 : 1), respectively, were only present in Saos-2 cells treated with PD alone or in combination with 2 Gy. Phospholipids are the primary components of the plasma membrane. Some of them can act as secondary messengers including phosphoinositides, which are derived from phosphoinositols. Phosphoinositols are in turn involved in intracellular signaling cascades following the activation of specific phospholipases, such as phospholipase C (PLC) in mammalian cells [31].

## 4. Discussion

Osteosarcomas are bone tumors occurred principally in children or adolescents [32, 33]. Unfortunately, the presence of mesenchymal component at high-rate proliferation can induce metastasis and relapse and novel therapies need to be urgently discovered [34, 35]. Clinical treatment with polydatin is available for the therapy of patients with liver diseases in China but the knowledge of pathway trigged by its action inside the cells is still unclear. Polydatin has shown potential therapeutic applications due to its antiosteoporotic and anti-inflammatory activities; furthermore, it promotes the osteogenesis of human bone marrow stromal cells via the BMP2-Wnt/*β*-catenin pathway. In the present study, we have demonstrated that the PD is able to affect the osteosarcoma cell proliferation by inducing cell cycle arrest and a different localization of *β*-catenin. Moreover, at 48 *μ*M the PD showed no antiproliferative effect on mesenchymal stem cells (data not shown). The effect of PD on OS cell morphology suggests that the migration may also be negatively affected, likely involving EMT through the Wnt/*β*-catenin pathway.

**Figure 9:**
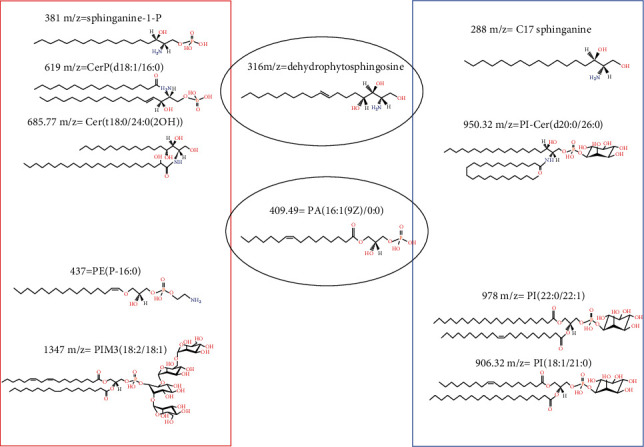
Sphingolipids secreted in the medium of PD-treated (red square) and PD-untreated Saos-2 cells (blue square). The sphingolipids indicated with black circles were commonly expressed in all assessed experimental conditions.

We also showed that pretreatment with polydatin sensitizes Saos-2 cells to ionizing radiation. As reported by Schwarz et al., radiotherapy is an important option as a local treatment of unresectable tumors or to avoid metastases. The probability of long-term survival, however, is low. In fact, the high radiation doses required to sterilize unresectable osteosarcomas are difficult to achieve with fractionated radiation therapy techniques [36]. By approaching OS as a disease treatable with a combination therapy using polydatin and radiation, we suggest developing novel therapies that can target OS differentiation and subsequently improve the effect of radiotherapy. Recent investigations show how osteogenic differentiation has a role in the pathogenesis of OS considering that OS tumors deregulate the signaling pathways associated with osteogenic differentiation by arresting the cells as undifferentiated precursors [37]. In this context, the expression of osteopontin (OPN) appears to be critical for the status of osteoblasts. OPN is necessary for modulating osteoblast differentiation through integrin *α*v*β*3-mediated cell signaling [38]. Although OPN overexpression has been associated with worse prognosis in many cancers, in osteosarcoma, the inhibition of OPN expression reduces the differentiation of mesenchymal stem cells or immature osteoblasts into mature osteoblasts thus leading to immature osteoblastic-like cells [30, 39]. Along with OPN, Notch signaling was important in osteogenic differentiation of adult stem cells and enhanced human periodontal ligament cells mineralization [40]. Our results proved that polydatin is a natural agent able to promote osteoblast-mediated mineralization and enhance the activity of bone phosphatase in osteosarcoma cells. Marrelli et al. [41] reported a case of adenomatoid odontogenic tumor (AOT) developed in an 18-year-old woman with familial adenomatous polyposis (aFAP). After surgical eradication and through the use of adjuvants of bone regeneration, a reconstruction of eroded bone tissue was performed using biotechnological products. Moreover, it was also reported that lymphoproliferative disorders can affect the oral cavity with possible involvement of the bone [42]. In these cases, the possible use of osteodifferentiating agents such as polydatin could be useful as adjuvant strategies for the bone reconstruction. In past years, evidence has accumulated that 2′-hydroxy ceramide/sphingolipids have distinct biological functions to regulate various cellular processes [43]. They also play a role in physical properties of cell membranes and regulate cell differentiation by binding to specific target proteins. Moreover, Sphingosine-1-phosphate (SP1) was reported to inhibit the osteoclast formation and the mineralization, respectively [22]. Finally, it was also reported that SP1 and its analogue FTY720 increase radiation sensitivity of human breast cancer cells *in vitro* [23]. Therefore, it can not be excluded that also in our cellular model the increase of SP1 levels induced by PD may act as a potentiating agent of anticancer effects of radiation therapy. In Figure 9, we have reported the scheme of the sphingolipid structures identified by mass spectrometry in the medium of PD-treated and PD-untreated Saos-2 cells. The production of the ceramides (619 *m*/*z* = Cer P(d18 : 1/16 : 0); 685.77 *m*/*z* = *Cer*(*t*18 : 0/24 : 0(2*OH*)) secreted in the medium of the PD-treated Saos-2 cells may be induced by de novo synthesis starting from serine and palmitoyl-CoA or by hydrolysis of sphingomyelin or glycosphingolipids (GSL). Therefore, on the basis of the identified structures, we hypothesize that PD induced a sphingolipid rheostat dynamic process suggesting new strategies for therapy of osteosarcoma based upon concomitant treatment of OS with radiotherapy and PD administration on a biological rationale.

## 5. Conclusions

OS is a tumor of bone which frequently develops metastases following treatments and is partially sensitive to radiotherapy. In the present study, we demonstrated that PD was able to induce cell cycle arrest in S phase, redistributed *β*-catenin protein, and enhanced osteoblast differentiation markers including BAP, osteopontin, and Notch2. We also found that PD sensitized Saos-2 cells to ionizing radiation, and this effect was paralleled by increased expression and secretion of ceramides and sphingolipids. The use of polydatin in combination with radiotherapy can consolidate the response to therapy of OS cells and can be used as an adjuvant added to radiotherapy for the local control of the disease and for the prevention of the local recurrence. Overall, our preliminary results suggest that PD may be considered a candidate for improving the efficacy of radiotherapy in patients with osteosarcoma and it is a starting point for additional studies on the mechanisms of OS radio-sensitization and PD activity.

## Figures and Tables

**Table 1:** The IC25 (*μ*M) values of PD on osteosarcoma cells.

	Saos-2	MG-63
24 h	122	Not determined
48 h	48	125
72 h	43	55

**Table 2:** Effect of polydatin on the percentage of cells in different stages of cell cycle Saos-2 cells as measured by flow cytometric analysis (^∗^*p* < 0.05; ^∗∗∗∗^*p* < 0.0001).

	G0/G1 (% of total)	S (% of total)	G2 (% of total)
72 h			
Saos-2	30.6 ± 6.96	12.7 ± 3.45	54.9 ± 9.91
Saos-2+PD	28.9 ± 7.88	^∗∗∗∗^26.9 ± 5.22	^∗^43.5 ± 10.0
96 h			
Saos-2	30.6 ± 7.52	8.3 ± 2.38	59.9 ± 10.1
Saos-2+PD	30.0 ± 7.02	^∗∗∗∗^22.5 ± 6.70	^∗^47.6 ± 10.50

## Data Availability

The data presented in this study are available on request from the corresponding author.
